# Yield of hybrid rye silage and its use as a replacement for barley silage on feed intake, growth performance, and carcass quality of growing and finishing steers

**DOI:** 10.1093/tas/txaf048

**Published:** 2025-04-14

**Authors:** Fuquan Zhang, Rebecca S Brattain, Herman Wehrle, Vern Baron, Gabriel O Ribeiro, Gregory B Penner

**Affiliations:** Department of Animal and Poultry Science, University of Saskatchewan, Saskatoon, Saskatchewan, S7N 5A8, Canada; KWS Cereals USA, LLC, Champaign, IL 61822, USA; KWS Seeds Canada, Calgary, Alberta T2T 0A4, Canada; AAFC-Lacombe Research and Development Centre, Lacombe, Alberta T4L 1W1, Canada; Department of Animal and Poultry Science, University of Saskatchewan, Saskatoon, Saskatchewan, S7N 5A8, Canada; Department of Animal and Poultry Science, University of Saskatchewan, Saskatoon, Saskatchewan, S7N 5A8, Canada

**Keywords:** feedlot cattle, growth performance, liver abscesses, silage

## Abstract

This study compared the yield of barley and hybrid rye when harvested for silage, and evaluated the effects of replacing barley silage (BARS) with hybrid rye silage (HRS) on dry matter intake (DMI), growth, and carcass characteristics of feedlot steers. The whole-plant hybrid rye (late milk) and barley (soft dough) were each harvested from 3 replicate 7.85-ha plots in 2022 and 2023 and ensiled (n = 3/treatment/yr). In each year, 192 steers were allocated to 1 of 16 pens in a completely randomized block design for growing (4 pens/treatment/yr) and finishing (5 or 6 pens/treatment/yr) phases. Treatments during the growing phase (84 d) included a control diet (GCON) that contained 60% BARS, and in the remaining treatments HRS replaced 33 (GLOW), 67 (GMED), or 100% (GHIGH) of the BARS (DM basis). Steers were then re-randomized and allocated to 1 of 16 pens for the finishing phase with diets that contained (DM basis) 10% BARS (FCON) or diets where HRS replaced 50% (FMED) or 100% (FHIGH) of the BARS. Steers were fed for 112 d. Forage DM yield did not differ between whole plant hybrid rye and barley (3.96 vs. 3.70 mT/ha). During the growing phase, increasing the HRS inclusion at the expense of BARS decreased DMI (quadratic, *P *= 0.02), average daily gain (ADG; quadratic, *P* < 0.01), and final BW (quadratic, *P* = 0.02) with the magnitude of the response increasing with increasing HRS inclusion. In addition, the gain:feed (G:F) ratio linearly decreased (*P *< 0.01) with increasing HRS inclusion. During finishing, DMI tended to linearly decrease (*P *= 0.06), ADG decreased and then increased (quadratic, *P *= 0.04) with the lowest ADG observed for FMED, while G:F was unaffected. Carcass weight for steers fed FMED and FHIGH did not differ but were lighter (quadratic, *P* = 0.02) than FCON, and dressing percentage decreased linearly from 58.81 to 58.34% (*P* = 0.03) as HRS inclusion increased. Carcass yield grade and marbling were unaffected. Increasing HRS inclusion at the expense of BARS linearly decreased the proportion of steers with minor (*P *= 0.02) and severe (*P *= 0.04) liver abscesses. In conclusion, while forage yield may not differ between hybrid rye and barley, increasing the inclusion rate of HRS in diets for growing steers reduced DMI and ADG. During finishing, increasing the inclusion of HRS may decrease DMI and resulted in lighter hot carcass weight without affecting carcass yield grade or marbling.

## Teaser

Fall planted hybrid rye yielded similar quantities of forage relative to spring planted barley but was harvested 1-mo earlier at an earlier stage of growth. Substituting barley silage with hybrid rye silage in growing and finishing diets for feedlot steers may decrease DMI, growth, and hot carcass weight without affecting carcass yield or marbling.

## INTRODUCTION

Whole plant silage from small cereal grains is an important forage source for beef cattle in the Northern Great Plains of the USA (Lemus, 2021; Sartin et al., 2022), in Western Canada (Baron and Kibite, 1987; Baron et al., 1992a), and in parts of Europe (Auerbach and Theobald, 2020). In fact, barley is one of the most common crops used for silage production in Western Canada and it has been recommended that whole plant barley is harvested at the soft dough stage of maturity to balance yield, quality, and to obtain an appropriate DM concentration for ensiling (Baron et al., 1992b; McAllister et al., 1995; Nair et al., 2016). However, changing weather patterns, interest in promoting the cover of soil with a living plant during winter, strategies to increase opportunity for crop rotation, and strategies to alter timing of ensiling have stimulated interest in other plant species (Magdoff and Van Es, 2021; SARE, 2023).

Cereal rye is a biennial plant that can be used for fall grazing in the year of seeding or early spring grazing following winter (Baron et al., 1999; IBC, 2018). In recent years, newer hybrid rye varieties have been introduced to North America from Europe. These fall planted hybrids show potential for greater yield, improved winter hardness, reduced ergot risk, increased drought tolerance, and have an earlier harvest date relative to spring planted barley (Geiger and Miedaner, 2009; McGhee and Stein, 2020; KWS, 2023). Cereal rye silage has been reported to have greater crude protein (CP) and neutral detergent fiber (NDF) concentrations, but less starch than barley silage (BARS) when harvested at mid-maturity (NASEM, 2021). It is important to note that the optimal harvest maturity of fall rye is at an earlier stage than for barley due to the rapid decline in quality and palatability after the heading stage of maturity (O’Reilly, 2024). Cereal rye forage was reported to have lower in vitro DM and NDF digestibility from the boot stage through the dough stage when compared with barley due to its greater concentrations of NDF, ADF, and lignin (Helsel and Thomas, 1987; Edmisten et al., 1998). However, to our knowledge, there are no published data comparing performance of cattle fed hybrid rye silage (HRS) as a replacement for BARS.

It was hypothesized that: 1) whole-plant hybrid rye harvested and ensiled at the late milk stage would provide greater DM yield and have greater NDF and lesser starch than that of whole-plant barley harvested and ensiled at the soft dough stage of maturity; 2) increasing HRS inclusion at the expense of BARS in diets for growing beef steers would decrease dry matter intake (DMI) and growth; and 3) increasing HRS inclusion in finishing diets as a replacement for BARS would not affect DMI, carcass quality, or the proportion of cattle with severe liver abscesses due to the relatively low silage inclusion rate. Thus, this study compared: 1) the yield and composition of whole plant hybrid rye and barley when harvested for silage; 2) DMI and growth for growing steers as HRS inclusion increased as a replacement of BARS; and 3) DMI, growth, carcass characteristics, and liver abscess scores for finishing steers fed diets with increasing inclusion of HRS as a replacement for BARS.

## MATERIALS AND METHODS

All experimental procedures involving the use of steers were approved by the University of Saskatchewan Animal Research Ethics Board (protocol 20210069) and followed the guidelines of the Canadian Council of Animal Care (Ottawa, ON, Canada). The experiment was conducted at the University of Saskatchewan Livestock and Forage Centre of Excellence (LFCE; Clavet, Saskatoon, SK, Canada).

### Study 1. Cereal Production

Six field plots with a mean ± SD area of 7.85 ± 0.301 ha were stratified by field and were randomly assigned to be seeded with either hybrid rye or barley silage (Clavet, SK, Canada) in each of 2 yr. Hybrid rye (*Secale cereale L*.; KWS Progas, FP Genetics, Regina, SK, Canada) was seeded at a rate of 98.6 kg/ha on August 18^th^ and 19^th^ in 2021 (yr 1), and 81.3 kg/ha on September 1^st^ and 2^nd^ in 2022 (yr 2). Differences in seeding rate for the hybrid rye were designed to achieve 1,980,000 seeds/ha based on the measured germination rate and thousand kernel weight. Barley (*Hordeum vulgare L*.; CDC Austenson, SeCan, Kanata, ON, Canada) was seeded at a rate of 134.5 kg/ha on May 13^th^ and 14^th^ in 2022 (yr 1), and on May 13^th^ in 2023 (yr 2). The soil was classified as Orthic Dark Brown Chernozemic soil formed in loamy lacustrine materials (Bradwell, SK, Canada; SKSIS Working Group, 2018). The hybrid rye plots were sprayed prior to seeding with glyphosate (Roundup Transorb HC; Bayer Cropscience Inc., Calgary, AB, Canada) at 1.66 L/ha and bromoxynil (Buctril M; Bayer Cropscience Inc., Calgary, AB, Canada) at 0.99 L/ha. Barley plots were sprayed 2 to 3 d before seeding with glyphosate at 0.86 L/ha (Roundup Transorb HC; Bayer Cropscience Inc., Calgary, AB, Canada) and dimethylamine salt at 0.47 L/ha (2,4-D Ester 700; Nufarm Canada, Calgary, AB, Canada). In addition, a nonionic penetrating surfactant was utilized with the herbicide spray for barley to reduce off-target spray drift to the adjacent hybrid rye plots. For barley plots, pyrasulfotole and bromoxynil (Infinity; Bayer Cropscience Inc., Calgary, AB, Canada), and prothioconazole and tebuconazole (Tilmor 240 EC Fungicide; Bayer Cropscience Inc., Calgary, AB, Canada) were sprayed 30 d after seeding at 0.84 L/ha, and 0.49 L/ha, respectively. In year 1, a granular fertilizer was used for HRS to provide 33.7 kg/ha N, 12.1 kg/ha P, 4.43 kg/ha K, and 3.0 kg/ha S. For barley, the granular fertilizer provided 71.6 kg/ha N, 45.7 kg/ha P, 11.4 kg/ha S, and 1.1 kg/ha Zn. For year 2, HRS fields were fertilized with granular fertilizer to provide 94.3 kg/ha N, 29.1 kg/ha P, 20.2 kg/ha K, and 10.7 kg/ha S, and for barley plots, granular fertilizer added 85.2 kg/ha N, 45.3 kg/ha P, and 12.8 kg/ha S. Fertilizer application rates were based on soil tests and recommendations from a local agronomist (Richardson Pioneer, Saskatoon, SK, Canada). Information on environmental data for each crop is reported in Table 1.

**Table 1.** Environmental conditions during crop growth for barley (*Hordeum vulgare L*.; CDC Austenson) and hybrid rye (*Secale cereale L*.; KWS Progas)^1^

Crop	Seeding date	Harvest date	Minimum temperature, °C	Mean temperature, °C	Maximum temperature, °C	Cumulative precipitation, mm^2^	Growing degree days^3^
Barley							
Year 1	13 May 2022	25 July 2022	−3.1	15.7	36.7	99.1	784.1
Year 2	13 May 2023	19 July 2023	1.6	17.7	32.9	111.9	836.2
Hybrid rye							
Year 1	18 August 2021	30 June 2022	−40.4	−0.5	32.9	165.8	988.8
Year 2	1 September 2022	22 June 2023	−38.2	−1.38	35.7	141.2	1036.5

^1^Date obtained from Environment and Climate Change Canada (2024) Saskatoon RCS station (https://climate.weather.gc.ca/historical_data/search_historic_data_e.html).

^2^The cumulative precipitation was calculated the total precipitation between the seeding and harvest dates.

^3^Growing degree days (GDD) was calculated as (maximum temperature + minimum temperature) ÷ 2—base, where base is 5 °C. If the GDD was <0, then it was set to 0 for that day.

In each replicate plot, the hybrid rye and barley were routinely monitored to assess the stage of maturity for harvest by visual evaluation according to the Zadoks growth scale (Zadoks et al., 1974). Hybrid rye was swathed and chopped for ensiling at the late milk stage on June 30^th^, 2022 and June 22^nd^, 2023. Barley was harvested at the soft dough stage of maturity on July 25^th^, 2022 and July 19^th^, 2023. Both crops were chopped to a theoretical chop length of 1.2 cm and were simultaneously mixed with an inoculant (Magniva Classic; Lallemand Animal Nutrition, Milwaukee, WI, USA) at a rate of 9.0 × 10^7^*Pediococcus pentosaceus* NCIMB 12455 colony forming units/kg (CFU/kg) and 1.0 × 10^7^*Lactobacillus plantarum* NCIMB 12422 CFU/kg of fresh silage. In each year, HRS was mechanically compacted using a silage bagger (AB1214 Ag-Bagger; Ag-Bag by RCI, Mayville, WI, USA), while BARS was mechanically compacted with a tractor and ensiled using a covered pile or a concrete bunker for year 1 and 2, respectively. The HRS and BARS were sealed beneath one layer of polythene plastic. The forage yield from each plot was recorded by weighing each truckload of fresh forage on a calibrated scale. Samples of fresh chopped forage were collected from each truckload at the time of packing and were used to generate 2 subsamples within a plot. One subsample was dried using a forced-air oven (55 °C) until achieving a constant weight for the determination of DM. The other subsample was frozen at −20 °C until being submitted for chemical analysis.

Silage samples were collected 2 mo after ensiling using a drill-driven probe (Star Quality Samplers Inc., Irricana, AB, Canada) prior to initiating the feedlot study. The green-chop samples and silage samples were vacuum sealed, frozen at −20 °C, and sent to Fraser Analytical Services (Abbotsford, BC, Canada) for chemical analysis using near-infrared spectroscopy (NIR). Silage samples collected throughout the feedlot study were also used for fermentation analysis using NIR at Fraser Analytical Services. Both the BARS and HRS were ensiled for 5 to 6 mo before being opened the day prior to the initiation of feedlot study.

### Study 2. Effect of Replacing Barley Silage with Hybrid Rye Silage for Growing Beef Steers

#### Animal management, experimental design, and dietary treatments.

This study was replicated in 2022 and 2023 with year considered as a blocking factor. At the beginning of the study in each year, a total of 192 steers were sourced from a local auction market [mean body weight (BW) of 326 ± 23.9 kg, and 359 ± 21.6 kg for years 1 and 2, respectively]. The processing protocols upon receiving, pen conditions, and bedding provided in pens (wheat straw) were the same as described by Zhang et al. (2024). Briefly, steers were provided a herd management tag, and vaccinated against bovine respiratory disease, infectious bovine rhinotracheitis, bovine viral diarrhea, parainfluenza type 3, histophilosis (Bovi-shield Gold One Shot; Zoetis Canada, Kirkland, QC, Canada), and clostridial diseases (Ultrabac 7/Somnubac; Zoetis Canada, Kirkland, QC, Canada). Steers were also treated for internal and external parasites (Bovimectin Pour-on; Bimeda Canada, Cambridge, ON, Canada) and were implanted with Ralgro upon arrival (36 mg zeranol; Merck Animal Health, Kirkland, QC, Canada). Steers were adapted to pens and feeding management using a common growing diet for 30 d prior to the start of the study. In year 1, the common diet consisted of dry-rolled barley grain 17%, barley silage 56.5%, oat hulls 20%, canola meal 6%, and limestone 0.5% (as fed basis). In year 2, steers received a common diet containing dry-rolled barley grain 13%, barley silage 57.5%, hay 13%, oat hulls 8%, canola meal 8%, and limestone 0.5% (as fed basis). For both years, the mineral and vitamin supplement was added using a micro-machine (Micro-Weigh System; Micro Technologies Feedlot Solutions, Amarillo, TX, USA). Steers were visually assessed daily by trained staff blinded to treatments, and steers presenting symptoms of illness were further assessed and treated as required.

At the start of the study in each year, steers were weighed on two consecutive days with the BW values from the first day of measurement used to stratify steers by BW. Within strata, steers were randomly assigned to 1 of 16 pens (4 pens per treatment/yr; 12 steers per pen/yr) in a completely randomized block design with pen as the experimental unit. Steers were re-implanted with 100 mg trenbolone acetate, 10 mg estradiol, and 29 mg of tylosin tartrate (Component TE 100; Elanco Canada Ltd., Mississauga, ON, Canada) on the day prior to the start of the study.

The control diet (Table 2) for year 1 contained 60.00% BARS, 38.43% barley grain, 1.43% limestone, 0.10% salt white, 0.02% urea, and 0.02% of a vitamin and mineral supplement (DM basis). Hybrid rye silage was used as a replacement for BARS and replaced 0.00% (GCON), 33.00% (GLOW), 67.00% (GMED), or 100.00% (GHIGH) of the barley silage. For year 2, the proportions of barley grain and urea changed slightly to maintain the same forage inclusion and similar dietary chemical composition between the 2 yr but the replacement of BARS with HRS remained constant. Steers were fed for 84 d in both years of the study. Total mixed rations were prepared using a horizontal mixer (Kuhn RC 260V feed mixer; Kuhn North America, Inc., Brodhead, WI, USA) mounted on a truck with a scale allowing for 4.5 kg of resolution. Cattle were fed once daily between 0830 and 1100 h, with the amount of feed adjusted to achieve ad libitum intake while minimizing residual feed in the bunk. As described above, a micro-machine (Micro-Weigh System; Micro Technologies Feedlot Solutions, Amarillo, TX, USA) was used to deliver trace minerals, vitamins, and feed additives into the mixer. All diets contained monensin to achieve a dietary concentration of 33 mg/kg on a DM basis (Rumensin Premix; Elanco Canada Ltd., Mississauga, ON, Canada). The treatment diets were formulated to meet or exceed the energy and protein requirements for growing cattle for targeting a final BW of 450 kg and an ADG of 1.3 kg/d (NASEM, 2016).

**Table 2.** Ingredients, chemical composition of experimental diets fed to growing steers in each of 2 yr

Item	Hybrid rye silage inclusion^1^							
	GCON		GLOW		GMED		GHIGH	
Year	1	2	1	2	1	2	1	2
n	3	3	3	3	3	3	3	3
Ingredient, % of DM								
Barley silage	60.00	60.00	40.20	40.20	19.80	19.80	-	-
Hybrid rye silage	-	-	19.80	19.80	40.18	40.20	60.00	60.00
Barley grain	38.43	38.29	38.24	38.34	38.07	38.39	37.87	38.44
Limestone	1.43	1.43	1.43	1.43	1.43	1.43	1.43	1.43
Salt white	0.10	0.10	0.10	0.10	0.10	0.10	0.10	0.10
Supplement^2^	0.02	0.02	0.02	0.02	0.02	0.02	0.02	0.02
Urea	0.02	0.16	0.21	0.11	0.40	0.06	0.58	0.01
Chemical composition, % of DM^3^								
DM, %	43.51 ± 0.449	44.05 ± 1.219	44.99 ± 0.580	45.56 ± 0.914	46.64 ± 0.772	47.23 ± 0.745	48.33 ± 1.009	48.99 ± 0.927
OM	92.14 ± 0.184	92.13 ± 0.214	92.46 ± 0.227	92.69 ± 0.179	92.79 ± 0.274	93.26 ± 0.172	93.12 ± 0.322	93.82 ± 0.198
CP	13.87 ± 0.162	13.21 ± 0.239	13.98 ± 0.351	12.97 ± 0.313	14.07 ± 0.552	12.72 ± 0.434	14.16 ± 0.748	12.47 ± 0.569
ADF	18.27 ± 0.292	17.75 ± 0.174	18.77 ± 0.162	18.57 ± 0.368	19.28 ± 0.287	19.42 ± 0.742	19.78 ± 0.500	20.24 ± 1.119
NDF	35.74 ± 0.408	35.00 ± 0.421	36.59 ± 0.579	35.76 ± 0.504	37.46 ± 0.671	36.53 ± 0.703	38.32 ± 0.941	37.29 ± 0.942
aNDFom	34.12 ± 0.180	33.03 ± 0.317	35.28 ± 0.418	34.14 ± 0.544	36.46 ± 0.671	35.29 ± 0.783	37.63 ± 0.918	36.40 ± 1.016
Starch	28.64 ± 0.826	26.46 ± 0.415	26.34 ± 0.801	25.29 ± 0.629	23.97 ± 0.803	24.08 ± 0.863	21.66 ± 0.830	22.90 ± 1.097
Ether extract	3.62 ± 0.204	5.25 ± 1.153	3.90 ± 0.137	4.98 ± 0.724	4.19 ± 0.270	4.70 ± 0.286	4.47 ± 0.448	4.43 ± 0.166
Ca	0.69 ± 0.019	0.66 ± 0.006	0.67 ± 0.020	0.66 ± 0.001	0.65 ± 0.021	0.67 ± 0.008	0.63 ± 0.022	0.68 ± 0.015
P	0.34 ± 0.058	0.24 ± 0.009	0.34 ± 0.058	0.25 ± 0.010	0.33 ± 0.049	0.26 ± 0.011	0.33 ± 0.044	0.26 ± 0.012
peNDF^4^								
peNDF_19.0_	0.78 ± 0.009	2.56 ± 0.031	0.86 ± 0.014	2.24 ± 0.031	0.94 ± 0.019	1.88 ± 0.036	1.02 ± 0.025	1.53 ± 0.039
peNDF_8.0_	10.07 ± 0.115	16.48 ± 0.198	12.81 ± 0.203	16.86 ± 0.237	15.75 ± 0.320	17.24 ± 0.332	18.74 ± 0.460	17.62 ± 0.445
peNDF_4.0_	18.57 ± 0.212	19.75 ± 0.238	19.59 ± 0.310	20.14 ± 0.284	20.66 ± 0.420	20.54 ± 0.395	21.75 ± 0.534	20.93 ± 0.528
NEm, Mcal/kg^5^	1.79 ± 0.013	1.91 ± 0.052	1.80 ± 0.022	1.89 ± 0.023	1.81 ± 0.038	1.87 ± 0.007	1.81 ± 0.055	1.85 ± 0.035
NEg, Mcal/kg^5^	1.18 ± 0.013	1.27 ± 0.049	1.18 ± 0.038	1.26 ± 0.025	1.19 ± 0.029	1.24 ± 0.002	1.19 ± 0.040	1.22 ± 0.025

^1^The control diet (GCON) contained 38.43 and 38.29% barley grain (year 1 and 2, respectively), 60% barley silage (both years), 1.43% limestone (both years), 0.10% salt white (both years), 0.02% vitamin and mineral supplement (both years). Urea was used to balance dietary CP among diets. Hybrid rye silage replaced 0, 33 (GLOW), 67 (GMED), and 100% (GHIGH) of the barley silage.

^2^Supplement contained: 7.65% Ca; 0.47% Mg; 0.03% K; 0.01% Na; 2.51% S; 100,071 mg/kg Mn; 50,036 mg/kg Cu; 805 mg/kg Fe; 150,106 mg/kg Zn; 4,289 mg/kg I; 652 mg/kg Co; 630 mg/kg Se; 33 mg/kg monensin (Elanco Animal Health, Greenfield, IN) on a DM basis.

^3^Chemical composition is expressed as means ± SD of the means.

^4^Physical effective NDF was calculated by multiplying the pef by the silage NDF content where pef was determined as the proportion of silage particles (as fed basis) retained on 19-, 8-, and 4-mm sieves. It was assumed that only silage contributed peNDF.

^5^Net energy values were calculated from the chemical composition of feed samples according to the equations of NRC (2001).

Throughout the study, samples of BARS and HRS were collected from the face of the silage twice weekly with duplicate samples collected on each sampling day. Barley grain was sampled once a week. The processing index (PI) of the barley grain (mean ± SD) was 69.7 ± 1.49% in year 1 (n = 15) and 70.5 ± 1.11% in year 2 (n = 17). The PI was calculated by dividing the 500-mL weight of the processed barley grain by the 500-mL weight of the unprocessed whole barley grain (Yang et al., 2000). Feed refusals were removed from each pen every 3 wk. The weight of the refusals was recorded, and a sample was collected. All feed ingredient and refusal samples were dried at 55 °C until achieving constant weight to determine the DM concentration. Dietary DM coefficients were adjusted on a weekly basis if the DM of the 3-wk running average differed by more than 2 percentage units than the existing coefficients. The weight difference between the amount of feed DM offered and amount of DM refused were used to determine pen DMI. Pen-level values were divided by the number of steers in the pen and by the number of days to determine the average DMI (kg/d). In addition, particle size distribution of the BARS and HRS samples were determined using the Penn State Particle Separator (Nasco, Fort Atkinson, Wisconsin, USA) with aperture sizes of 19-, 8-, and 4-mm, and a bottom pan using the method described by Heinrichs (2013).

Steers were weighed every 21 d prior to feeding, and weighing coincided with feed refusal removal and measurement. The BW data were regressed with the day of the study to calculate ADG. The unshrunk ADG was used to determine the gain-to-feed (G:F) ratio by dividing the ADG by DMI. At the end of growing phase, steers were weighed on two consecutive days to determine the final BW. The dietary net energy values for maintenance (NE_m_) and gain (NE_g_) were estimated based on growth performance. The retained energy (RE) for medium-framed yearling steer calves was used (RE = [0.0635 × EBW^0.75^] × EBG^1.097^), where empty body weight (EBW) = 0.891 × shrunk BW; empty body weight gain (EBG) = 0.956 × shrunk BW gain (NASEM, 2016). The shrunk BW was the shrunk (4% shrink) mid-test weight adjusted at 28% body fat using the equation: mid-test shrunk BW × (478 / final shrunk BW) (NASEM, 2016). The NE_m_ concentration in the diet was calculated using the quadratic formula (Zinn et al., 2002), and the NE_g_ was determined from NE_m_ according to Zinn and Shen (1998) using the equation: NE_g_ = NE_m_ × 0.877−0.41.

### Study 3. Effect of Replacing Barley Silage with Hybrid Rye Silage for Finishing Beef Steers

The end of study BW data collected from the growing phase were used to re-randomize steers into 1 of 16 pens/yr while ensuring to have an equal distribution of steers from each growing phase treatment in each pen. Pens were then randomly assigned to 1 of 3 treatments and were re-implanted with 200 mg trenbolone acetate and 28 mg estradiol benzoate (Synovex Plus; Zoetis Canada, Kirkland, QC, Canada) at the start of the study. Dietary treatments (Table 3) included the control diet (FCON) that contained 10% silage (DM basis), and diets where HRS replaced 50% (FMED), or 100% (FHIGH) of the BARS (DM basis). The remainder of the diets included 87.78% barley grain, 1.97% limestone, 0.22% salt white, and 0.03% supplement. In year 1, 5 pens were assigned to FCON, 5 pens to FMED, and 6 pens to FHIGH, while in year 2, 5 pens were allocated to FCON, 6 pens to FMED, and 5 pens to FHIGH. Steers were fed for 112 d in each year. The finishing diets were formulated to supply sufficient energy, protein, and minerals and vitamins to meet or exceed predicted requirements of beef steers with a final BW of 650 kg and a ADG of 2.0 kg/d (NASEM, 2016). Steers were transitioned to their respective finishing diets over 25 d with the transition consisting of 5 step-up diets with each diet fed for 5 d (Supplementary Table 1). In each step-up diet, the amount of grain increased as a replacement for silage. The final treatment diets were fed on d 26 and the dietary transition was included in the performance results. Feed and refusal collection, and TMR preparation and delivery were conducted as described in Study 2. The DMI, ADG, G:F, as well as net energy values for finishing phase were calculated as previously described in Study 2.

**Table 3.** Ingredients, chemical composition of experimental diets fed to finishing steers in each of 2 yr

	Hybrid rye silage inclusion^1^					
Item	FCON		FMED		FHIGH	
Year	1	2	1	2	1	2
n	2	2	2	2	2	2
Ingredient, % of DM						
Barley silage	10		5		-	
Hybrid rye silage	-		5		10	
Barley grain	87.78		87.78		87.78	
Limestone	1.97		1.97		1.97	
Salt white	0.22		0.22		0.22	
Supplement^2^	0.03		0.03		0.03	
Chemical composition, % of DM^3^						
DM, %	77.73 ± 0.854	76.61 ± 1.114	79.16 ± 0.932	76.94 ± 0.790	80.65 ± 0.585	77.28 ± 0.492
OM	94.76 ± 0.316	95.05 ± 0.023	94.89 ± 0.328	95.08 ± 0.068	95.03 ± 0.340	95.12 ± 0.114
CP	13.96 ± 0.167	12.09 ± 0.516	13.87 ± 0.142	12.19 ± 0.562	13.78 ± 0.117	12.29 ± 0.608
ADF	8.45 ± 1.103	7.95 ± 0.752	8.60 ± 1.117	8.25 ± 0.738	8.76 ± 1.131	8.55 ± 0.724
NDF	21.56 ± 1.124	20.04 ± 1.979	21.85 ± 1.047	20.31 ± 1.866	22.14 ± 0.969	20.58 ± 1.753
aNDFom	20.92 ± 1.442	19.33 ± 1.841	21.31 ± 1.371	19.69 ± 1.781	21.70 ± 1.300	20.08 ± 1.721
Starch	49.83 ± 3.428	50.15 ± 0.238	49.24 ± 3.417	49.84 ± 0.284	48.64 ± 3.407	49.53 ± 0.330
Ether extract	2.87 ± 0.068	3.11 ± 0.196	2.88 ± 0.125	3.07 ± 0.085	2.89 ± 0.181	3.04 ± 0.026
Ca	0.73 ± 0.003	0.73 ± 0.017	0.73 ± 0.002	0.73 ± 0.014	0.73 ± 0.001	0.74 ± 0.011
P	0.33 ± 0.025	0.27 ± 0.038	0.34 ± 0.024	0.28 ± 0.037	0.34 ± 0.023	0.28 ± 0.037
peNDF						
peNDF_19.0_	0.08 ± 0.004	0.24 ± 0.024	0.09 ± 0.004	0.19 ± 0.018	0.10 ± 0.004	0.14 ± 0.012
peNDF_8.0_	1.01 ± 0.053	1.57 ± 0.155	1.40 ± 0.067	1.60 ± 0.147	1.80 ± 0.079	1.62 ± 0.138
peNDF_4.0_	1.87 ± 0.097	1.89 ± 0.186	1.98 ± 0.095	1.91 ± 0.175	2.10 ± 0.092	1.92 ± 0.164
NEm, Mcal/kg^5^	2.06 ± 0.000	2.09 ± 0.035	2.05 ± 0.002	2.08 ± 0.028	2.05 ± 0.005	2.07 ± 0.021
NEg, Mcal/kg^5^	1.41 ± 0.002	1.43 ± 0.035	1.41 ± 0.002	1.42 ± 0.029	1.40 ± 0.005	1.41 ± 0.023

^1^The control diet (FCON) contained 87.78 % barley grain, 10% barley silage, 1.97 % limestone, 0.22% salt white, 0.03% vitamin and mineral supplement. Hybrid rye silage replaced 0, 50 (FMED), and 100% (FHIGH) of the barley silage.

^2^Supplement contained: 9.18% Ca; 0.57% Mg; 0.03% K; 0.02% Na; 3.01% S; 120,085 mg/kg Mn; 60,044 mg/kg Cu; 966 mg/kg Fe; 180,127 mg/kg Zn; 5,147 mg/kg I; 783 mg/kg Co; 756 mg/kg Se; 33 mg/kg monensin (Elanco Animal Health, Greenfield, IN) on a DM basis.

^3^Chemical composition is expressed as means ± SD of the means.

^4^Physical effective NDF was calculated by multiplying the pef by the silage NDF content where pef was determined as the proportion of dietary silage particles (as fed basis) retained on 19-, 8-, and 4-mm sieves. It was assumed that dietary NDF was only contributed from silage sources.

^5^Net energy values were calculated from the chemical composition of the feed samples according to the equations of NRC (2001).

### Feed Analysis

Dried feed samples were ground to pass through a 1-mm screen using a hammer mill (Christie-Norris Laboratory Mill, Christie-Norris Ltd, Chelmsford, UK). The ground feed ingredient samples were composited on an equal weight basis by month resulting in 3 samples/yr/ingredient in Study 2. For Study 3, 2-mo composites were prepared resulting in 2 samples for each feed ingredient that were submitted for chemical analysis. Subsequently, the composited feed samples were sent to Cumberland Valley Analytical Services (Waynesboro, PA, USA) for analysis of analytical DM, organic matter (OM), CP, NDF, NDF corrected for ash content (aNDFom), acid detergent fiber (ADF), starch, ether extract, calcium, and phosphorus as described by Delver et al. (2023).

### Carcass Characteristics

At the end of Study 3, all steers were fed in the morning, then loaded at 1300 h and transported 660 km to a federally inspected abattoir (Cargill Meat Solutions, High River, AB, Canada). Steers were held overnight at the abattoir with access to water but no access to feed. Two researchers, blinded to treatments, audited the slaughter process to confirm steer and carcass sequence, and to assess liver scores. Steers were euthanized by captive bolt stunning, exsanguinated, and the hide, head, feet, internal organs, and kidney, pelvic, and heart fat along with the respective organs were removed prior to measurement of hot carcass weight (HCW). The HCW was used to determine dressing percentage (DP) by dividing the carcass weight by the average final BW measured at the end of the study after correction (4%) for shrink. Carcass-adjusted final BW was calculated for each pen as the average HCW divided by the average DP for the whole population. The back-fat thickness, ribeye area, yield score, marbling percentage, and marbling score between the 12^th^ and 13^th^ ribs were determined using the Computer Vision Grading System (VBG 2000 e + v Technology GmbH, Oranienburg, Germany). Liver scores were evaluated using the method of Brown and Lawrence (2010) with modifications to include three classifications according to the Elanco Liver Check System (Elanco Animal Health, Greenfield, IN, USA).

### Statistical Analysis

All data were analyzed using the Mixed model of SAS (SAS version 9.4; SAS Institute, Cary, NC, USA). The crop yield and chemical composition data were analyzed using the PROC GLIMMIX procedure as a randomized complete block design with the fixed effect of silage type, and the random effects of year and plot nested within year. The Kenward-Roger method for calculating degrees of freedom was used. Least squared means (LSMEANS) were computed for the fixed effect of silage type.

Data for growth performance (BW, ADG, DMI, and G:F in Study 2 and 3) and carcass traits (HCW, DP, back-fat thickness, rib eye area, yield score, marbling percentage, and marbling score in Study 3) were analyzed using the GLIMMIX procedure of SAS including the fixed effect of dietary treatment and the random effect of year within block. Liver scores were analyzed using GLIMMIX procedure of SAS with binominal error structure and logit data transformation. The covariance structure for the random effects was specified as variance components. The LSMEANS option and Kenward-Roger method to calculate degrees of freedom were used. The model was fit using the Newton-Raphson Ridge (NRRIDG) optimization technique to ensure convergence. A maximum of 1000 iterations was allowed to facilitate model convergence. The significance of covariance effects was tested using Wald tests. Polynomial contrasts were used to determine whether linear, quadratic (Study 2 and 3), and cubic (Study 2 only) responses occurred. For all data analysis, significant differences between means were declared when *P* ≤ 0.05, and tendencies were considered when 0.05 < *P* ≤ 0.10.

In Study 2, 1 steer on the GHIGH treatment was found dead due to bloat in yr 2. In Study 3, 2 steers on the FCON treatment were removed from the study (one for lameness and swelling in the stifle joint, and the other due to polioencephalomalacia) in yr 1. In yr 2, 3 steers fed the FCON diet were removed from Study 3. One was found dead due to bloat, and the other 2 were pulled out of study as they did not respond to treatment for lameness. All the steers that did not complete the study were excluded for data analysis.

## RESULTS

### Study 1. Crop Production and Chemical Composition of Whole Crop Barley and Hybrid Rye

Total precipitation throughout the growing season for hybrid rye and barley were 166 and 99 mm (yr 1), and 141 and 112 mm (yr 2), respectively (Table 1). However, the precipitation values were below the 12-yr historical average (2009 to 2021) during the growing months (hybrid rye and barley, respectively) for the region of 244 ± 97.8 mm, and 178 ± 89.4 mm (mean ± SD; Environment and Climate Change Canada, 2024). There were no differences for the wet and DM yields between whole plant barley when harvested at the soft dough stage and whole plant hybrid rye harvested at the late milk stage (*P*≥ 0.19; Table 4). The hybrid rye forage had greater DM, OM, ADF, NDF, and aNDFom concentrations than barley forage (*P*≤ 0.04). In contrast, CP, starch, and ether extract concentrations were less for hybrid rye than barley forage (*P*≤ 0.01). The concentrations of Ca and P did not differ (*P* ≥ 0.24). When factoring yield and chemical composition, OM yield did not differ (*P* = 0.10), hybrid rye produced greater ADF, NDF, and aNDFom yields than that of barley (*P *< 0.03). In contrast, starch yield for barley was greater than that for hybrid rye (*P *= 0.02). The CP and ether extract yields did not differ.

**Table 4.** Yield, chemical composition of whole-plant barley (Hordeum vulgare L.; CDC Austenson) and hybrid rye forage (Secale cereale L.; KWS Progas) over 2 yr^1^

Item	Barley	Hybrid rye	SEM^2^	*P*-value
n	6	6		
Stage of maturity	soft dough	late milk		
Area, ha	7.85	7.85		
Forage yield, kg wet/ha	12,030	10,517	2,307.3	0.19
Forage yield, kg DM/ha	3,699	3,956	749.3	0.22
Chemical composition, % DM				
DM, %	31.44	37.56	0.751	<0.01
OM	93.46	95.02	0.503	0.04
CP	13.03	11.08	0.467	0.01
ADF	23.95	27.67	0.546	<0.01
NDF	45.35	48.67	0.540	<0.01
aNDFom	43.78	48.05	0.596	<0.01
Starch	11.20	3.07	0.783	<0.01
Ether extract	3.61	2.97	0.154	0.01
Ca	0.27	0.23	0.022	0.24
P	0.23	0.23	0.018	0.91
Yield, kg/ha				
OM	3432.0	3754.2	689.94	0.10
CP	491.9	420.1	90.73	0.32
ADF	902.7	1110.5	209.47	0.01
NDF	1681.3	1933.3	359.43	0.03
aNDFom	1625.1	1911.3	353.17	0.01
Starch	432.9	112.8	76.64	0.02
Ether extract	135.0	116.1	26.39	0.35

^1^In each year, the whole-plant barley and hybrid rye were sampled and composited from each truckload within each plot (n = 3/treatment/year) at ensiling and then analyzed for chemical composition using NIR.

^2^Greatest SEM is presented.

### Study 2. Effect of Replacing Barley Silage with Hybrid Rye Silage for Growing Beef Steers

The DM, OM, ADF, NDF, and aNDFom concentrations of HRS were numerically greater than that of BARS (Table 2) used throughout Study 2 and 3 across the 2 yr, while the starch content of HRS was numerically lower than that of BARS (Table 5). As this was an ingredient substitution study, increasing the proportion of HRS as replacement of BARS in growing diets numerically increased the dietary DM, aNDFom, and ADF concentrations while reducing the starch concentration. All silages had a mean pH ≤ 4.2 with lactic acid being the predominant acid (≥72% of the total acid) suggesting appropriate ensiling characteristics.

**Table 5.** Dry matter, chemical composition, fermentation products, and particle size distribution of whole-plant barley silage and hybrid rye silage^1^

Item	Barley silage		Hybrid rye silage	
	Year 1	Year 2	Year 1	Year 2
n	5	5	5	5
Nutrient content, % DM				
DM, %^2^	31.29 ± 5.037	33.53 ± 1.802	36.23 ± 5.835	36.65 ± 3.057
OM	91.52 ± 0.424	91.70 ± 0.611	94.11 ± 0.458	93.54 ± 0.611
CP	12.70 ± 0.453	13.08 ± 0.691	10.80 ± 0.616	13.53 ± 0.904
ADF	27.44 ± 0.838	25.58 ± 0.602	30.22 ± 1.221	30.46 ± 1.698
NDF	48.50 ± 0.453	46.48 ± 2.364	53.50 ± 1.606	51.82 ± 2.321
aNDFom	46.06 ± 0.358	45.17 ± 0.850	52.78 ± 1.771	50.90 ± 3.051
Starch	13.02 ± 0.471	8.74 ± 0.971	1.60 ± 0.200	2.64 ± 0.503
Ether extract	4.45 ± 0.403	6.41 ± 1.710	5.41 ± 0.834	5.28 ± 0.701
Ca	0.25 ± 0.072	0.25 ± 0.026	0.20 ± 0.030	0.31 ± 0.028
P	0.23 ± 0.048	0.20 ± 0.005	0.27 ± 0.017	0.23 ± 0.011
NE_m_, Mcal/kg	1.63 ± 0.029	1.78 ± 0.092	1.64 ± 0.077	1.64 ± 0.085
NE_g_, Mcal/kg	1.03 ± 0.025	1.15 ± 0.082	1.03 ± 0.059	1.04 ± 0.078
Particle size distribution, %^4^				
>19.0 mm	3.63 ± 0.997	12.19 ± 3.155	4.45 ± 2.544	6.82 ± 2.073
<19.0 > 8.0 mm	43.31 ± 4.103	66.30 ± 9.649	77.05 ± 2.297	71.94 ± 2.629
<8.0 > 4.0 mm	39.65 ± 4.020	15.57 ± 9.520	13.11 ± 1.949	14.77 ± 2.325
<4.0 mm	13.40 ± 2.149	5.94 ± 1.457	5.39 ± 0.987	6.47 ± 1.158
Ensiling characteristics				
n	3	3	3	3
pH	3.8 ± 0.30	3.8 ± 0.04	3.9 ± 0.14	4.2 ± 0.04
Total organic acids, % DM	10.1 ± 2.53	13.9 ± 2.10	8.3 ± 0.21	6.9 ± 0.37
Lactic acid, % DM	8.8 ± 2.42	10.4 ± 0.35	6.0 ± 0.86	6.3 ± 0.29
Acetic acid, % DM	1.3 ± 0.11	3.5 ± 1.97	2.4 ± 0.64	0.6 ± 0.16

^1^The values are expressed as means ± SD of the means.

^2^The DM content was calculated as the average of values from samples collected on a weekly basis throughout growing and finishing phases in each of 2 yr.

^3^The particle size distribution was calculated as the average of values from samples collected on a weekly basis throughout growing and finishing phases in each of 2 yr.

While initial BW did not differ among treatments, increasing HRS inclusion quadratically decreased final BW with an increasing magnitude as HRS inclusion increased (*P* = 0.02; Table 6). Dry matter intake, regardless if reported as kg/d or as a percentage of BW was quadratically affected such that the reduction in intake increased as the inclusion of HRS increased (*P*≤ 0.03). In addition, ADG (quadratic, *P* < 0.01), and G:F (linear, *P* < 0.01) decreased as HRS inclusion increased as a replacement for BARS. Increasing the inclusion of HRS at the expense of BARS resulted in linear reductions for dietary NEm (*P* = 0.01) and NEg (*P* = 0.01) concentrations.

**Table 6.** Effect of feeding hybrid rye silage as a replacement for barley silage on feed intake, and growth performance of steers during growing phase over 2 yr

Item	Hybrid rye silage inclusion^1^				SEM^2^	*P*-value			
	GCON	GLOW	GMED	GHIGH		Treatment	Linear	Quadratic	Cubic
n	8	8	8	8					
Initial BW, kg	343	342	343	343	10.0	0.81	0.97	0.93	0.34
Final BW, kg	462	456	450	432	9.3	<0.01	<0.01	0.02	0.17
DMI, kg/day	9.23	9.10	8.82	8.08	0.196	<0.01	<0.01	0.02	0.61
DMI, % of BW	2.29	2.29	2.23	2.09	0.031	<0.01	<0.01	0.03	0.85
ADG, kg/day^3^	1.45	1.36	1.30	1.08	0.044	<0.01	<0.01	<0.01	0.10
G:F, kg/kg	0.158	0.150	0.147	0.134	0.0056	<0.01	<0.01	0.20	0.06
NEm, Mcal/kg^4^	1.81	1.76	1.77	1.74	0.037	0.05	0.01	0.52	0.25
NEg, Mcal/kg^4^	1.17	1.14	1.14	1.12	0.032	0.06	0.01	0.56	0.31

^1^The control diet (GCON) contained 38.43 and 38.29% barley grain (year 1 and 2, respectively), 60% barley silage (both years), 1.43% limestone (both years), 0.10% salt white (both years), 0.02% vitamin and mineral supplement (both years). Urea was used to balance dietary CP among diets. Hybrid rye silage replaced 0, 33 (GLOW), 67 (GMED), and 100% (GHIGH) of the barley silage.

^2^Greatest SEM was reported.

^3^ADG was calculated by regressing BW with the days on feed throughout the experiment.

^4^Net energy values were determined based on animal growth performance as described by Zinn et al. (2002) and Zinn and Shen (1998). The shrunk BW was the shrunk (4% shrink) mid-test weight adjusted at 28% body fat (NASEM, 2016).

### Study 3. Effect of Replacing Barley Silage with Hybrid Rye Silage for Finishing Beef Steers

As described above, the two silage sources used in this study were the same as Study 2. Thus, substitution of BARS with HRS numerically increased the DM, OM, ADF, and aNDFom, concentrations, whereas the starch concentration decreased slightly (Table 3).

By design, initial BW was not affected by treatment, but final BW responded quadratically (*P* < 0.01; Table 7) such that BW was at least 10 kg greater for FCON than either FMED or FHIGH. Dry matter intake (kg/day) tended to decrease linearly as the inclusion of HRS increased as a replacement for BARS (*P* = 0.06); however, there was no effect of treatment when DMI was represented as a percentage of BW (*P* = 0.49). Average daily gain (quadratic, *P* = 0.04) decreased and then increased with increasing inclusion of HRS. Despite differences in DMI and ADG, G:F (*P* = 0.62) was not affected by HRS inclusion rate. Carcass-adjusted final BW responded quadratically (*P* = 0.03) as it decreased from FCON to FMED with no difference between FMED to FHIGH. There was a quadratic (*P* = 0.03) response for carcass-adjusted ADG where ADG decreased from FCON to FMED and then increased for FHIGH. Increasing HRS as replacement of BARS had no effect on carcass-adjusted G:F, or on the performance-based estimates for NEg and NEm during the finishing phase.

**Table 7.** Effect of feeding hybrid rye silage as a replacement of barley silage on feed intake, and growth performance of growing steers during finishing phase

Item	Hybrid rye silage inclusion^1^			SEM^2^	*P*-value		
	FCON	FMED	FHIGH		Treatment	Linear	Quadratic
n	10	11	11				
Initial BW, kg	451	447	447	8.9	0.33	0.19	0.47
Final BW, kg	657	646	647	10.5	<0.01	<0.01	<0.01
DMI, kg/day	11.46	11.20	11.23	0.172	0.08	0.06	0.18
DMI, % of BW	2.07	2.05	2.06	0.021	0.63	0.49	0.49
ADG, kg/day^3^	1.89	1.82	1.86	0.040	0.09	0.33	0.04
G:F, kg/kg	0.165	0.163	0.166	0.0030	0.36	0.62	0.19
Carcass-adjusted final BW, kg^4^	633	619	619	10.6	<0.01	<0.01	0.03
Carcass-adjusted ADG, kg/day	1.83	1.74	1.77	0.040	0.02	0.06	0.03
Carcass-adjusted G:F	0.160	0.156	0.158	0.0028	0.21	0.41	0.12
NEm, Mcal/kg^5^	1.84	1.83	1.85	0.020	0.38	0.30	0.36
NEg, Mcal/kg^5^	1.20	1.20	1.21	0.018	0.40	0.28	0.43

^1^The control diet (FCON) contained 87.78% barley grain, 10% barley silage, 1.97% limestone, 0.22% salt white, 0.03% vitamin and mineral supplement. Hybrid rye silage replaced 0, 50 (FMED), and 100% (FHIGH) of the barley silage.

^2^Greatest SEM was reported.

^3^ADG was calculated by regressing BW with the days on feed throughout the experiment.

^4^Carcass-adjusted BW was determined by using the hot carcass weight divided by the total average dressing percentage of 58.86 and 58.15% (year 1 and 2, respectively), and then used to calculate ADG and G:F.

^5^Net energy values were determined based on animal growth performance as described by Zinn et al. (2002) and Zinn and Shen (1998). The shrunk BW was the shrunk (4% shrink) mid-test weight adjusted at 28% body fat using the equation: mid-test shrunk BW × (478 / final shrunk BW). The empty body weight (EBW) and empty body weight gain (EBG) were calculated using equations: EBW = 0.891 × shrunk BW; EBG = 0.956 × shrunk BW gain (NASEM, 2016).

Increasing HRS inclusion rate decreased (quadratic, *P* = 0.02) hot carcass weight by 9 kg for both FMED and FHIGH relative to FCON (Table 8). Dressing percentage decreased linearly by 0.48 percentage units (FCON to FMED) and 0.47 percentage units (FCON to FHIGH) as HRS replaced BARS (*P* = 0.03) but backfat thickness, ribeye area, yield score, marbling percentage, and marbling score were not affected by HRS inclusion. The proportions of cattle without liver abscesses linearly increased with increasing HRS inclusion rate (*P* < 0.01) and the proportion of cattle with minor (*P* = 0.02) and severe liver abscesses (*P* = 0.04) linearly decreased with increasing inclusion of HRS when used as a replacement for BARS in finishing diets.

**Table 8.** Effect of feeding hybrid rye silage as a replacement of barley silage on carcass characteristics and liver abscesses of growing steers during finishing phase

Item	Hybrid rye silage inclusion^1^			SEM^2^	*P*-value		
	FCON	FMED	FHIGH		Treatment	Linear	Quadratic
n	10	11	11				
Hot carcass weight, kg	371	362	362	5.9	<0.01	<0.01	0.02
Dressing percentage, %	58.81	58.33	58.34	0.207	0.05	0.03	0.19
Back fat thickness, cm	1.35	1.33	1.35	0.058	0.95	0.99	0.74
Ribeye area, cm^2^	92.93	91.83	92.27	1.677	0.65	0.58	0.46
Yield score	2.89	2.85	2.84	0.121	0.89	0.65	0.89
Marbling, %	2.93	2.90	2.89	0.121	0.90	0.66	0.92
Marbling score^3^	395.8	397.5	384.8	6.59	0.31	0.23	0.35
Liver score, %^4^							
Clear	72.11	73.34	77.92	4.290	0.01	<0.01	0.28
Minor	4.32	3.79	2.34	0.837	0.05	0.02	0.38
Severe	23.56	22.84	19.76	3.566	0.10	0.04	0.43

^1^The control diet (FCON) contained 87.78% barley grain, 10% barley silage, 1.97% limestone, 0.22% salt white, 0.03% vitamin and mineral supplement. Hybrid rye silage replaced 0, 50 (FMED), and 100% (FHIGH) of the barley silage.

^2^Greatest SEM was reported.

^3^United States Department of Agriculture (USDA) marbling scores: 200 to 299 = trace; 300 to 399 = slight; 400 to 499 = small; 500 to 599 = modest; and 600 to 699 = moderate.

^4^Liver scores were classified as clear (0 to 1 or 2 small abscesses), minor (2 to 4 small active abscesses), and severe (1 or more large active abscesses) as adapted from Brown and Lawrence (2010).

## DISCUSSION

### Forage Yield

Although previous field studies have suggested that HRS has greater forage yield than spring seeded barley when both crops were harvested at boot stage (KWS, 2023), we did not observe differences for wet or DM yields. The lack of a yield response was unexpected, as fall rye is recognized as a drought tolerant forage due to its ability to utilize soil moisture in late winter and early spring (Clark, 2008). It is important to recognize that part of the response for the lack of yield between hybrid rye and barley may be related to differences in the stage of maturity at the time of harvest and consequently the harvest date. In the present study, we harvested hybrid rye at the late milk stage of maturity while barley was harvested at a more advanced stage of maturity (soft dough). This resulted in harvest of hybrid rye approximately 1 mo prior to harvest of barley silage. The stage of maturity selected for barley was based on previous recommendations for balancing yield and chemical composition (Kaulbars and King, 2004; Nair et al., 2018); whereas, hybrid rye was harvested at an earlier stage of maturity due to its potential for a rapid decline in digestibility (Helsel and Thomas, 1987; Coblentz et al., 2000) and reduced palatability (O’Reilly, 2024) with advancing maturity. As such, while this study did not attempt to balance yield estimates at the same stage of maturity, the stages utilized are reflective of those adopted under commercial production settings for each crop. As such, based on similar yield and an earlier date of harvest, use of hybrid rye as a silage source may offset risk associated growing season conditions.

Another possible reason for the lack of DM yield difference in this study between hybrid rye and barley may be due to drought condition. Drought stress is typically the primary constraint on forage yield (Buxton and Fales, 1994; Moore et al., 2020). The total precipitation for hybrid rye and barley were 79 and 78 mm (yr 1), and 66 and 103 mm (yr 2), respectively less than the 12-yr average precipitation during the same growing months. When combining precipitation with the date harvest for the two crops, the drought conditions could have constrained yield of hybrid rye. However, Baron et al. (2015) reported that two cultivars of fall rye (15.2 and 12.4 t/ha) yielded numerically greater than that of two spring barley cultivars (12.0 and 11.5 t/ha) when both crops were harvested at the dough stage in central Alberta, Canada. Thus, the hybrid fall rye in the present may have had greater DM yield than that of barley if both of crops were harvested at the same stage of maturity. However, as noted above, harvest maturities selected were chosen based on recommended stages and practices of producers in western Canada.

Although forage yield did not differ, forage harvested for HRS had greater concentrations of DM, OM, aNDFom, ADF, and less starch and CP than BARS. The NASEM (2021) reported that conventional rye silage had greater CP and NDF, but less starch than barley when both crops are harvested at mid-maturity stage. The observed greater NDF, ADF, and lower starch concentrations for HRS relative to BARS in the present study were in agreement with NASEM (2021) and Helsel and Thomas (1987); while, the lower CP concentration for HRS was unexpected. It is widely recognized that CP concentration decreases with advancing maturity for cereal crops (Cherney and Marten, 1982; Helsel and Thomas, 1987; Khorasani et al., 1997) and the rate of decline for CP of rye occurs rapidly (Helsel and Thomas, 1987; Kantar et al., 2011). In contrast, the CP concentration of barley decreases rapidly from heading until the boot stage, but declines slowly from the milk to soft dough stages (Khorasani et al., 1997; Nair et al., 2018). Thus, it is possible that low CP concentration for HRS was due to a rapid decline in concentration and that this decline was more rapid than occurred for barley, despite being harvested at a later stage of maturity. However, it is possible that part of the CP response is cultivar specific as when harvested at the same stage of maturity, CP concentrations varied by more than 3 percentage units for two rye cultivars and 9 percentage units for two barley varieties across two geographic locations in a 2-yr study (Helsel and Thomas, 1987). Cultivar variation along with differences in maturity are further supported as it has been reported that CP concentration of hybrid rye forage varieties can be as high as 17.35% at the boot stage of maturity but may drop to 8.51% at the soft dough stage maturity when seeded and harvested across the United States (KWS, 2023).

### Study 2. Effect of Silage Sources on Growth Performance during Growing Phase

To the authors knowledge, this is the first study evaluating the use of hybrid rye as a silage source for feedlot cattle. In the present study, increasing HRS as replacement for BARS in diets for growing cattle decreased DMI, ADG, G:F, and final BW with the magnitude of effect increasing as HRS inclusion increased. It is possible that the greater aNDFom concentration within HRS relative to BARS, and consequently greater dietary aNDFom with increasing HRS inclusion may have contributed to the DMI decrease (Allen, 2014, 2023; Oba and Kammes-Main, 2023). For example, relative to GCON, increasing HRS as a replacement for BARS increased the dietary NDF concentration by 0.85 percentage units for GLOW, 1.72 percentage units for GMED, and by 2.58 percentage units for GHIGH in yr 1. In yr 2, the NDF concentration increased by 0.76 percentage units for GLOW, 1.53 percentage units for GMED, and 2.29 percentage units for GHIGH relative to GCON. Consistent with the present study, a meta-analysis of 18 experiments with dairy cows indicated that DMI decreased with increasing dietary NDF content from 22.5 to 45.8% (Arelovich et al., 2008); a range consistent with the observed NDF concentrations in the present study. In addition to NDF concentration, NDF digestibility can alter filling effects of NDF. Helsel and Thomas (1987) reported that the in vitro DM disappearance for rye cultivars harvested at milk and soft dough stages were lower than those of barley cultivars. In addition, Keles et al. (2016) observed that DMI and live BW gain of lambs fed fresh rye forage harvested at the milk stage were less than for lambs fed barley harvested at the dough stage. While not evaluated in the present study, it is possible that HRS had lesser DM and NDF digestibility, even when harvested at earlier stage of maturity than the BARS (Helsel and Thomas, 1987; Keles et al., 2016). If true, lesser digestibility may have contributed to a longer retention time in the rumen and hence a greater rumen filling effect (Allen, 2000). Support for this statement is provided by the linear reduction in G:F and the NEm and NEg concentrations with increasing HRS inclusion. Thus, it may be possible that rumen fill, driven in part by lower digestibility, limited DMI leading to compromised growth and feed efficiency. Future studies evaluating HRS should include diets that are formulated for equal NDF concentration instead of using equal dietary inclusion rates as used in this study.

It is also possible that differences in palatability may have contributed to differences in DMI. For dairy cattle, it is recommended to harvest cereal rye for silage at the flag-leaf or early boot stage of maturity because of the rapid decline in quality (Edmisten et al., 1998), palatability, and negative impact on feed intake (O’Reilly, 2024) after the heading stage. However, feedlot producers in western Canada have adopted a late-milk stage of maturity for harvest timing, similar to that used in the present study, to capture greater DM yield. Despite this practice, Lardy et al. (2022) reported that the palatability of rye hay rapidly declines following the flowering stage and the HRS in the present study was harvested at the late milk stage implying that perhaps harvesting earlier may avoid such reductions in DMI. More advanced maturity may lead to a more pronounced palatability response to awns present on the rye spikelet. Rye awns have a greater proportion of thick-walled cells, mostly as sclerenchyma tissue, relative to barley awns (Evert, 2006; Ntakirutimana and Xie, 2019) and presence of these awns may provide a mechanism for reduced palatability helping to explain the reduction in DMI observed for cattle fed HRS than BARS.

While the reduction in growth is likely driven by reduced DMI and potentially altered NDF digestibility, including HRS as a replacement for BARS also resulted in a dilution of the starch concentration with a magnitude of reduction greater than the increase in NDF. As such, part of the reduction for the NEm and NEg concentrations and ADG are likely due to the dilution of starch, particularly given that starch concentrations for BARS were 11.2 and 6.1 percentage units higher than that of HRS in yr 1 and yr 2, respectively. As such, when compounded over the course of the 84-d growing phase in each year, the lesser starch for HRS and consequently the treatments containing HRS may have resulted in decreased energy intake and subsequently decreased ADG and G:F.

### Study 3. Effect of Silage Sources on Growth Performance and Carcass Characteristics during the Finishing Phase

We hypothesized that increasing HRS as a replacement for BARS in finishing diets would not affect DMI, ADG, or G:F due to the low inclusion rate of silage in finishing diets. However, despite the subtle differences in dietary starch and NDF, increasing inclusion of HRS tended to linearly reduce DMI resulting in lower final BW and a 9 kg reduction in carcass weight for treatments including HRS than for FCON. Galyean and Defoor (2003) and Arelovich et al. (2008) summarized data from published studies evaluating the effects of roughage NDF source and dietary NDF, respectively, on DMI in high-concentrate diets fed to feedlot cattle. In contrast with the present study, DMI generally increased as NDF supplied by roughage increased and with increasing dietary NDF concentration. The increase in DMI with increased NDF concentration is thought to occur through energy dilution given that ruminal fill likely does not contribute much to regulation of DMI for finishing cattle (Galyean and Defoor, 2003; Koenig et al., 2020). However, dietary NEm and NEg values calculated based on feed intake and growth were not different challenging whether HRS inclusion diluted the energy density of the diet. As noted above for Study 2, it is possible that the tendency for a reduction in DMI and consequently the reductions for final BW and carcass weight could be related to palatability of HRS relative to BARS (Clark, 2008; Lardy et al., 2022), particularly since the NEg and NEm values did not differ. Potential reasons for altered palatability were discussed above for Study 2.

We are unaware of other studies evaluating carcass characteristics and the incidence of liver abscesses for steers fed HRS. The current results are consistent with previous studies where hot carcass weight and dressing percentage were positively associated with greater final BW (Bruns et al., 2004; Wilken et al., 2015; Sperber et al., 2024). However, there were no differences for back fat thickness, ribeye area, yield grade, marbling percentage, or marbling score further suggesting that the reduction for HCW was likely driven by reduced DMI. Although the contribution of dietary forage NDF to energy supply for finishing feedlot cattle is small, forages varying in fiber composition, peNDF, and uNDF affect ruminal motility, eating behavior, ruminal fermentation, ruminal SCFA absorption, as well as total tract nutrient digestibility (Pereira et al., 2023a, 2023b). Others have also reported that despite low inclusion, the forage source impacts performance of finishing cattle (Johnson et al., 2020; Pereira et al., 2021; Carey et al., 2023). As such, it is plausible that in addition to DMI, differences in digestibility of the BARS and HRS may have contributed to altered carcass weight observed in the present study (Owens et al., 1995; Liu et al., 2024).

The proportion of steers with liver abscesses (minor and severe) decreased with increasing inclusion of HRS as replacement of BARS. Liver abscesses in feedlot cattle have been reported to be a polymicrobial disease where pathogens pass through the damaged ruminal or intestinal epithelial barrier caused by ruminal acidosis, ruminitis, or other factors impacting gastrointestinal barrier function (Nagaraja and Lechtenberg, 2007; Hales, 2024). Using the classical theory, the reduction in liver abscesses may suggest that increasing HRS inclusion mitigated ruminal acidosis incidence as a mechanism to reduce the proportion of cattle with minor and severe liver abscesses. A meta-analysis conducted by Zellmer (2021) investigated the effect of source and concentration of forage NDF on DMI, growth performance, carcass characteristics, and liver abscesses. The authors noted that a higher forage NDF concentration in feedlot diets was associated with reduced incidence of liver abscesses. Additionally, it should be noted that the concomitant tendency for reduced DMI in the present study may partially contribute to the lesser proportion of liver abscesses where decreased DMI would have decreased the supply of fermentable OM to the rumen. Forage NDF fractions in finishing diets varying in uNDF and peNDF components also differ in their capacity to stimulate DMI, chewing activity, rumination, and ruminal motility (Pereira et al., 2023a, 2023b). In agreement with the latter statement, the peNDF_4.0_ fraction increased as HRS replaced BARS in the finishing diets which may have stimulated rumination, saliva production, and ruminal contractions helping to stabilize ruminal pH. Thus, the positive effect of HRS towards the mitigation of minor and severe liver abscesses suggests that further characterization of HRS NDF fractions may be warranted for steers fed high concentrate diets.

## CONCLUSION

These data indicate that barley and hybrid rye, when harvested at the soft dough and late milk stages, respectively could be expected to have similar DM yields with hybrid rye having lesser starch but greater NDF. However, hybrid rye harvest occurs approximately 1 mo prior to that for barley allowing for earlier forage availability and altered risk for forage production and preservation. Use of HRS as a replacement for BARS in diets for growing steers decreased DMI, ADG, and G:F with the magnitude of effect increasing as HRS inclusion increased. In addition, there was a tendency for a linear reduction in DMI, and quadratic responses for final BW, hot carcass weight, and dressing percentage such that diets containing HRS had lesser values than when BARS was fed as the sole forage. As such, while hybrid rye may yield similarly to barley, use of HRS may reduce feed intake and growth for growing and finishing cattle and reduce carcass weight. However, the results also indicate a trade-off between the compromised feed intake and concomitant growth, and the reduced incidence of liver abscesses with increasing HRS inclusion rate.

## Supplementary Material

txaf048_suppl_Supplementary_Table_S1
