# Supplementary material for: Yield of hybrid rye silage and its use as a replacement for barley silage on feed intake, growth performance, and carcass quality of growing and finishing steers
Source: Transl Anim Sci. 2025 Apr 14;9:txaf048. doi: 10.1093/tas/txaf048 (PMC12057561; doi:10.1093/tas/txaf048)
Supplement: txaf048_suppl_Supplementary_Table_S1 [file txaf048_suppl_supplementary_table_s1.docx]

Supplement Table 1. Ingredient composition of the diets fed to steers during transition phase from growing diets to finishing diets over 25 d across 2 years^1^

|  | Stage of transition | | | | | |
| --- | --- | --- | --- | --- | --- | --- |
| Ingredient, % DM | Step 1 | Step 2 | Step 3 | Step 4 | Step 5 | Final |
| FCON |  |  |  |  |  |  |
| Barley silage | 60.00 | 48.00 | 38.00 | 28.00 | 19.00 | 10.00 |
| Hybrid rye silage | - | - | - | - | - | - |
| Barley grain | 38.43 | 50.43 | 60.43 | 69.78 | 78.78 | 87.78 |
| Limestone | 1.43 | 1.43 | 1.43 | 1.97 | 1.97 | 1.97 |
| Salt white | 0.10 | 0.10 | 0.10 | 0.22 | 0.22 | 0.22 |
| Supplement^2^ | 0.02 | 0.02 | 0.02 | 0.03 | 0.03 | 0.03 |
| Urea | 0.02 | 0.02 | 0.02 | - | - | - |
| FMED |  |  |  |  |  |  |
| Barley silage | 30.00 | 24.00 | 19.00 | 14.00 | 9.50 | 5.00 |
| Hybrid rye silage | 30.00 | 24.00 | 19.00 | 14.00 | 9.50 | 5.00 |
| Barley grain | 38.43 | 50.43 | 60.43 | 69.78 | 78.78 | 87.78 |
| Limestone | 1.43 | 1.43 | 1.43 | 1.97 | 1.97 | 1.97 |
| Salt white | 0.10 | 0.10 | 0.10 | 0.22 | 0.22 | 0.22 |
| Supplement^2^ | 0.02 | 0.02 | 0.02 | 0.03 | 0.03 | 0.03 |
| Urea | 0.02 | 0.02 | 0.02 | - | - | - |
| FHIGH |  |  |  |  |  |  |
| Barley silage | - | - | - | - | - | - |
| Hybrid rye silage | 60.00 | 48.00 | 38.00 | 28.00 | 19.00 | 10.00 |
| Barley grain | 38.43 | 50.43 | 60.43 | 69.78 | 78.78 | 87.78 |
| Limestone | 1.43 | 1.43 | 1.43 | 1.97 | 1.97 | 1.97 |
| Salt white | 0.10 | 0.10 | 0.10 | 0.22 | 0.22 | 0.22 |
| Supplement^2^ | 0.02 | 0.02 | 0.02 | 0.03 | 0.03 | 0.03 |
| Urea | 0.02 | 0.02 | 0.02 | - | - | - |

^1^Each step of the transition phase was 5 d in duration with the final finishing diets being fed on day 26.

^2^Supplement contained: 9.18% Ca; 0.57% Mg; 0.03% K; 0.02% Na; 3.01% S; 120,085 mg/kg Mn; 60,044 mg/kg Cu; 966 mg/kg Fe; 180,127 mg/kg Zn; 5,147 mg/kg I; 783 mg/kg Co; 756 mg/kg Se; 33 mg/kg monensin (Elanco Animal Health, Greenfield, IN) on a DM basis.
